# Online Food Purchase Frequency Across Generations: Associations with Healthy Eating, Environmental Concern, and Economic Decision Indicators

**DOI:** 10.3390/nu18162730

**Published:** 2026-08-20

**Authors:** Robert Wolny, Marcin Awdziej, Magdalena Jaciow, Ilona Lipowska, Marcin Lipowski, Jolanta Tkaczyk

**Affiliations:** 1 Department of Digital Economy Research, Faculty of Economics, University of Economics in Katowice, 40-287 Katowice, Poland; magdalena.jaciow@ue.katowice.pl; 2 Department of Marketing, Kozminski University, 03-301 Warszawa, Poland; mawdziej@kozminski.edu.pl (M.A.); jtkaczyk@kozminski.edu.pl (J.T.); 3 Department of IT Systems and Logistics, Faculty of Economics, Maria Curie-Sklodowska University, 20-031 Lublin, Poland; ilona.lipowska@mail.umcs.pl; 4 Department of Marketing, Faculty of Economics, Maria Curie-Sklodowska University, 20-031 Lublin, Poland; marcin.lipowski@mail.umcs.pl

**Keywords:** online food purchasing, generational cohorts, healthy eating, environmental concern, economic decision indicators, e-consumers

## Abstract

Background/Objectives: Online food purchasing may be associated with health-related, environmental, economic, and technological factors. However, limited evidence exists on whether these factors are associated with online food purchase frequency in similar ways across generational groups. This study examined whether self-reported healthy eating, environmental concern, and economic decision indicators were associated with online food purchase frequency among Polish consumers who had previously purchased food online and whether these associations differed across birth-cohort groups. Methods: Data were collected in Poland in 2025 in a cross-sectional study using Computer-Assisted Web Interviewing. The analytical sample comprised 669 respondents who declared having purchased food online. Online food purchasing was measured both as a binary distinction between frequent and occasional purchasing and as an eight-category ordinal frequency variable. Pearson’s chi-square tests, Mann–Whitney U tests, binary logistic regression, and ordinal logistic regression were applied. Moderation was assessed through predictor-by-generation interaction terms. The Benjamini–Hochberg procedure was used to control the false discovery rate, and seven economic decision-making indicators were additionally examined in mutually adjusted multivariable models. Ordinal models were interpreted as sensitivity analyses because the proportional-odds assumption was not fully satisfied. Results: Healthy eating was not directly associated with online food purchasing frequency and did not interact significantly with generation. Environmental concern showed a significant interaction with generation in both binary and ordinal models. It was associated with more frequent online food purchasing among Baby Boomers but with less frequent purchasing among Generation Z. In separate models, price comparison and careful product choice were associated with more frequent online purchasing. Saving orientation also showed an exploratory interaction with opposite directions across generational groups: it was associated with more frequent purchasing in Generation X and less frequent purchasing in Generation Z. However, these economic decision indicators did not remain robust after correction for multiple testing and simultaneous adjustment. Conclusions: Online food purchasing frequency was not uniformly associated with health, environmental, or economic tendencies across generations. In this cross-sectional sample, generation moderated the association between environmental concern and online food purchase frequency, although this pattern should not be interpreted as evidence of a distinct causal cohort effect. The findings suggest that similar declared tendencies may be associated with different patterns of online food purchase frequency across generational groups.

## 1. Introduction

The rapid development of digital technologies and e-commerce has fundamentally transformed consumer behaviour. The Internet has evolved from being merely a source of information into a key channel for purchasing products and services. Consumers increasingly use digital technologies to search for product information, compare offers, and complete purchases, while the boundaries between traditional and online retail continue to blur [1,2,3]. These changes are particularly evident in the food market. The expansion of e-commerce platforms, mobile applications, and home delivery services has made food one of the fastest-growing product categories in online retail. Digital channels provide convenient access to product offers, detailed information, and opportunities to compare alternatives. At the same time, food remains a unique product category because purchasing decisions are influenced not only by price and availability but also by health, quality of life, and environmental considerations.

In recent years, healthy eating has become an increasingly important determinant of consumer behaviour. Growing awareness of the relationship between diet and health has encouraged consumers to pay greater attention to product composition, nutritional value, and potential health benefits. Previous studies indicate that health is now among the most influential factors affecting food choice [4,5]. At the same time, environmental issues related to food production and consumption have gained increasing importance. Consumers are becoming more aware of the environmental consequences of their dietary choices and are increasingly incorporating sustainability considerations into their purchasing decisions [6,7,8]. Nevertheless, economic factors remain important determinants of food choice, even among consumers with strong health or environmental concerns [4,8]. Consequently, food purchasing decisions often reflect a trade-off between health, environmental, and economic considerations [5,9], highlighting the importance of examining these dimensions simultaneously.

Consumers, however, do not constitute a homogeneous group. They differ in their values, life experiences, digital competencies, and attitudes towards health, environmental protection, and financial management. One commonly used approach to describing this heterogeneity is the comparison of generational groups. Previous research has identified differences among generational groups in values, attitudes, technology use, and consumption patterns. However, in cross-sectional research, such differences may reflect not only cohort-related experiences but also age, life stage, household circumstances, and familiarity with digital technologies [10,11,12]. These differences are particularly evident in the adoption of digital technologies but may also extend to healthy eating, environmental concern, saving behaviour, preferred information sources, and the criteria used when selecting products and retailers.

Despite the growing body of research on food purchasing behaviour, several important research gaps remain. Existing studies have predominantly focused on individual aspects of consumer behaviour, such as healthy eating, environmental concern, economic determinants of food choice, or online grocery shopping. Comparatively little attention has been paid to integrating these dimensions within a single analytical framework. Consequently, limited evidence exists on whether healthy eating, environmental concern, and economic decision-making indicators are jointly associated with online food purchase frequency and whether these associations differ across generational groups.

The aim of this study is to examine whether healthy eating, environmental concern, and economic decision indicators are associated with online food purchasing frequency among Polish consumers who have previously purchased food online and whether these associations differ across generational cohorts. Accordingly, the study addresses the following research questions:

RQ1. To what extent does the frequency of online food purchasing differ among Baby Boomers, Generation X, Generation Y, and Generation Z?

RQ2. Are healthy eating and environmental concern directly associated with the frequency of online food purchasing?

RQ3. Does generational cohort moderate the relationships of healthy eating and environmental concern with online food purchasing frequency?

RQ4. Which economic decision indicators—saving orientation, budget control, deferred purchase, expense tracking, price comparison, shopping planning, and careful product choice—are directly associated with online food purchasing frequency?

RQ5. Does generational cohort moderate the relationships between economic decision-making and online food purchasing frequency?

RQ6. Which associations remain statistically independent when all economic decision indicators are examined simultaneously and generation is controlled?

The paper consists of theoretical and empirical sections and focuses on differences in online food purchasing frequency across generational groups. Section 1 introduces the research background and rationale. Section 2 reviews the theoretical foundations of healthy eating, environmental concern, online food purchasing behaviour, and economic decision-making styles and presents the conceptual research model. Section 3 describes the research methodology. Section 4 presents the empirical findings concerning differences in online food purchase frequency across the analysed generational groups. Section 5 discusses the results, while Section 6 outlines their practical implications for organizations operating in the food market. Section 7 addresses the limitations of the study and suggests directions for future research. Finally, Section 8 presents the main conclusions.

## 2. Theoretical Foundations of Online Food Purchasing

### 2.1. Healthy Eating in Food Purchasing

Healthy eating orientation refers to the importance consumers attach to maintaining a healthy diet when evaluating and selecting food products. It is reflected in attention to nutritional value, ingredients, product composition, and the potential health consequences of food choices [5,13]. This food-specific orientation should be distinguished from general health orientation or health consciousness, which encompasses a broader concern for personal health and health-related behaviour. Consumers are becoming increasingly health-conscious and therefore tend to select food products that not only provide nutritional benefits but also contribute to psychological satisfaction, while avoiding options perceived as potentially detrimental to their health [14]. Those with higher food-related competencies are more likely to incorporate health considerations into their food choices, demonstrating a stronger health orientation during food purchasing [15]. Azlie et al. [16] propose that attitudes toward health play a significant role in shaping food-buying intentions. More health-conscious consumers generally show greater sensitivity to attributes of health-related products, which leads to increased intentions to purchase healthier food choices. In societies where awareness of environmental issues and health-focused lifestyles is still developing, the influence of social circles and peers tends to be less pronounced. This results in weaker societal pressure to buy healthy or organic food products, which can lead to decreased intentions among consumers to make purchases in this category [17].

Health orientation can be defined as a consumer’s predisposition to prioritize health considerations in food-related decisions. It involves a preference for healthy foods, concern for personal well-being, and the belief that dietary choices can contribute to health maintenance and disease prevention [18]. It can further be understood as a long-term tendency to prioritize health in food choices, reflected in preferences for green products based on perceived benefits [19]. Szymkowiak et al. [9] distinguish between health orientation and healthy eating orientation as related but conceptually distinct constructs. Whereas health orientation refers to a broad concern for personal health and health-related behaviour, healthy eating orientation is more specific and reflects the importance individuals attach to maintaining a healthy diet when evaluating and selecting food products. The present study focuses on the latter construct. The concept of health orientation is closely associated with health consciousness [18], which can be understood as an individual’s awareness of health concerns, reflected in the degree to which health-related considerations influence everyday behaviours and choices [20]. As a key component of health orientation, it involves positive attitudes toward health, a commitment to preventive actions, and a willingness to adopt behaviours that support long-term physical and psychological well-being. It reflects people’s perspectives and opinions regarding their health issues and their readiness to prioritize their well-being [21].

Previous studies have associated health consciousness with food-related attitudes, attention to health-related product attributes, intentions to purchase healthier foods, and willingness to pay for selected health and environmental certifications [22,23,24,25,26]. These findings primarily concern product evaluation and selection rather than the frequency of using a particular purchasing channel. Recent findings indicate that skills related to food and knowledge of sustainable food practices (sustainable food literacy) are linked to healthier eating habits. This suggests that consumers who prioritize health are more inclined to turn their health concerns into practical dietary choices [27]. Health-conscious consumers often depend on product labels when assessing food items. They actively seek and process nutritional information when evaluating food products [28]. The previous literature has also shown that sustainability attributes influence consumers’ choices across different food categories [29]. At the same time, broader changes in food consumption patterns suggest that health and sustainability concerns may compete with other factors, such as convenience and accessibility. Research from various worldwide settings shows a significant shift in the eating habits of young people, as traditional food consumption increasingly gives way to dependence on ultra-processed and convenience foods [30]. Research from population-based studies shows significant differences in dietary habits among young consumers, revealing that both healthy and unhealthy eating practices can be present within this demographic, indicating varying degrees of health orientation regarding food choices [30]. A prevalent notion is that “unhealthy = tasty”, indicating that people view healthiness and flavour as being in opposition, with nutritious foods regarded as incompatible with the pursuit of pleasure [31].

### 2.2. Environmental Concern in Food Purchasing

Environmental concern has become an increasingly important determinant of food-related decision-making as consumers become more aware of the environmental consequences of food production, distribution, packaging, and consumption [32]. Defined as the degree to which individuals recognize environmental problems and support efforts aimed at their mitigation, environmental concern has been consistently linked to sustainable consumption behaviours, including preferences for organic, local, seasonal, and environmentally friendly food products [33,34,35]. The urgent societal and ecological need for a global transition toward sustainable diets has further strengthened interest in understanding how environmental values shape consumer food choices [36]. As food retailing increasingly shifts toward digital channels, understanding how environmental concern influences online food purchasing behaviour has become particularly relevant. Recent evidence suggests that purchasing food online may significantly affect food choice processes, dietary behaviour, and the evaluation of sustainability-related product attributes, making digital food environments an important context for studying environmentally responsible consumption [37].

Purchasing food online provides consumers with access to extensive product information, sustainability labels, certification schemes, and environmental claims. While foundational research in brick-and-mortar stores indicated that consumers often overlook ecological markers due to time constraints and limited visibility at the physical shelf [38], digital food retail environments create unique opportunities for environmentally concerned consumers to systematically incorporate sustainability considerations into their purchasing decisions. Recent studies suggest that online grocery platforms can facilitate more informed decision-making by enabling consumers to actively compare products based on attributes such as product origin, packaging sustainability, ethical sourcing practices, and environmental footprint information [39,40]. Research conducted in online grocery contexts further indicates that consumers increasingly rely on digital information cues when evaluating both nutritional and sustainability characteristics of food products [37]. Consequently, environmental concern may exert a stronger influence on food purchasing decisions in online settings than in conventional shopping environments where access to such detailed information is structurally limited.

However, the association between environmental concern and online food purchase frequency may differ across generational groups. The Generational Cohort Theory proposes that individuals exposed to similar social, economic, and cultural conditions during their formative years may develop partly shared values and consumption patterns [10]. Previous studies have reported that younger generational groups, particularly Generation Z and Millennials, tend to express higher levels of environmental awareness and stronger support for sustainable consumption than older groups [41,42]. Environmental challenges such as climate change, plastic pollution, biodiversity loss, and food waste may therefore be particularly relevant to the expectations that members of these groups express toward brands, retailers, and food producers [42,43].

Among Generation Z consumers, environmental concern has been associated with personal identity, ethical consumption, and social responsibility [42,43]. Previous studies also suggest that some members of this generational group use digital technologies to obtain and evaluate sustainability-related product information before purchase. In online food retailing, such information may include production methods, environmental certifications, packaging characteristics, and carbon-footprint indicators [44]. Digital environments can facilitate access to these cues, although their use and importance may vary considerably within Generation Z. Studies further suggest that younger consumers may expect retailers to communicate sustainability initiatives transparently and provide accessible environmental information at the point of purchase [45,46]. Previous studies have reported relatively strong environmental concern among Millennials, although their purchasing decisions may reflect trade-offs between sustainability considerations and practical factors such as convenience, price, and product availability [34,35]. Some findings also suggest that Millennials may be more willing to pay a premium for sustainable food products when environmental attributes are accompanied by personal benefits, including perceived healthfulness, food safety, nutritional quality, and product authenticity [35,47]. Environmental and health-related considerations may therefore jointly contribute to food-product evaluation. Within the broader framework of sustainable diets, personal health and environmental objectives are increasingly considered interrelated elements of consumer decision-making [36]. In contrast, some studies suggest that members of Generation X and the Baby Boomer group may place relatively greater emphasis on product quality, trust, familiarity, and economic value than on environmental attributes alone [48]. Among older consumers, the association between environmental concern and purchasing decisions may be more conditional. Environmentally sustainable food choices may be more likely when environmental attributes are accompanied by perceived functional benefits, such as freshness, local origin, superior quality, or health-related benefits [49,50]. Environmental concern among older generational groups may therefore form part of a broader product evaluation rather than constitute an independent purchasing motive. The digital context introduces additional complexity into these relationships. Previous studies have reported age-related differences in digital literacy and familiarity with online environments, including the use of online product comparisons and sustainability information [42,44]. Some older consumers may experience difficulties in evaluating the credibility of environmental claims presented online, potentially increasing information overload or scepticism toward sustainability-related marketing communications [51]. Digital competence may therefore be relevant to the association between environmental concern and the use of sustainability information in online food purchasing. Despite growing environmental awareness, the literature consistently highlights the existence of an attitude–behaviour gap in sustainable consumption. Consumers frequently express concern about environmental issues but do not always translate these concerns into actual purchasing behaviour [52,53]. This discrepancy may be particularly pronounced in online food retailing, where environmentally friendly options compete directly with lower-priced alternatives and where convenience often becomes a dominant decision criterion. Research on online grocery shopping suggests that consumers regularly face trade-offs between environmental preferences, health objectives, delivery convenience, packaging requirements, and economic considerations [37]. Such trade-offs may weaken the association between environmental concern and purchasing behaviour, especially among highly price-sensitive consumers.

Furthermore, environmental concern rarely operates in isolation. Food purchasing decisions are typically influenced by multiple motivations, including healthy eating, food safety concerns, ethical considerations, and economic decision styles [35,50]. This multidimensional nature of food choice is particularly relevant in online grocery shopping, where consumers are exposed to large volumes of information and must simultaneously evaluate nutritional, environmental, and economic product attributes. Consequently, environmental concern should be viewed as an important but context-dependent determinant of online food purchasing behaviour whose influence may vary across generations due to differences in values, digital competencies, and purchasing priorities. This perspective aligns with modern multidisciplinary research emphasizing that food choices are increasingly shaped by the simultaneous consideration of health, environmental sustainability, and economic constraints within digitally mediated food environments [36,37].

Overall, the literature suggests that environmental concern may be associated with online food purchasing, but this relationship is unlikely to be uniform across generational groups. Differences in environmental values, technology use, information-processing styles, and decision-making priorities may contribute to variation in how declared environmental concern relates to online food purchase frequency. Examining this variation is important for understanding the relationships between environmental concern, healthy eating, economic decision-making, and online food purchase frequency.

### 2.3. Online Food Purchasing Frequency

The frequency of online food purchasing may be associated with a range of situational, psychological, and technological factors [54]. Proliferations of these factors highlight the complexity of consumer decision-making in digital food retail environments [55].

Online food environments provide consumers with access to product descriptions, nutritional labels, retailer information, and user reviews. Because consumers cannot inspect products directly, the availability and credibility of these information cues may be relevant to product evaluation and purchase decisions [56]. However, access to information does not necessarily imply more frequent use of the online purchasing channel. When considering online food purchasing behaviour and information sources, it is essential to take into account label orientation. Consumers depend on product label information to assess health-related attributes and make informed purchasing decisions. Label orientation enhances consumers’ understanding of the health effects of their diet [13]. Individuals who prioritize health are more vigilant about product labels, ingredients, and nutritional details [29]. Recent studies show that clean-label indicators, like natural components, no artificial additives, and concise ingredient lists, improve perceived health benefits and boost purchase intentions [57]. Research on food packaging suggests that label orientation and label design significantly influence how consumers process product information and evaluate health-related attributes, thereby shaping food purchasing decisions [58].

While access to product information can facilitate informed food choices, excessive amounts of information may create additional difficulties for consumers. This phenomenon is commonly referred to as information overload [59]. Information overload can be defined as a situation in which the amount of available information exceeds an individual’s or an organization’s processing capacity, making it difficult to effectively identify and prioritize key information that should be communicated or acted upon [60]. As a result of the complexity involved in selecting healthy food, the average consumer often relies on heuristics, typically defaulting to repeatedly choosing the same familiar product within a matter of seconds [61]. Chen et al. [62] indicated that the simultaneous communication of health-oriented attributes and domestic origin may fail to produce synergistic perceptual effects due to information overload, which can reduce consumers’ ability to effectively integrate multiple product cues.

Recent research shows that shoppers choose online food retailers based on a blend of economic, functional, and informational factors. Important criteria include competitive pricing, convenience, delivery choices, ease of use of the platform, the variety of products, trust in the retailer, and availability of comprehensive product information. Furthermore, health- and sustainability-related considerations may be associated with consumers’ selection of online shopping channels [37,63,64].

### 2.4. Economic Decision-Making in Food Purchasing

Economic considerations constitute one of the most influential determinants of food purchasing behaviour and play a central role in shaping dietary choices across populations. Although consumers increasingly express interest in healthy and sustainable food consumption, economic constraints continue to influence the extent to which such preferences can be translated into actual purchasing behaviour [65,66]. While macro-level nutritional and environmental guidelines emphasize the urgent need for a global dietary shift [36], individual consumers must constantly reconcile these societal ideals with personal financial realities [67]. Within consumer behaviour research, economic decision styles refer to relatively stable patterns of evaluating and selecting products based on economic criteria, including price consciousness, value-for-money orientation, promotion sensitivity, budget management, and trade-offs between competing product attributes. Although originally conceptualized within general consumer contexts [68], the adaptation of these decision styles to food purchasing research highlights their profound influence not only on product selection but also on consumers’ willingness to prioritize nutritional quality, environmental sustainability, convenience, and product quality under strict budget constraints.

The importance of economic considerations in food purchasing has been extensively documented in both nutrition and consumer behaviour literature. Numerous studies identify food prices as one of the strongest predictors of food choices and dietary quality [65,66,69]. Historically, healthier dietary patterns have been frequently associated with higher food expenditures, while less nutritious, energy-dense diets often provide lower costs per calorie, creating substantial barriers to the adoption of recommended dietary behaviours, particularly among economically constrained consumers [65,66]. Similarly, sustainable and environmentally friendly food products, including organic and locally sourced foods, often carry price premiums that may discourage their purchase despite positive consumer attitudes toward sustainability [35,50].

Consequently, consumers frequently face food choice trade-offs involving affordability, nutritional quality, convenience, sensory appeal, and environmental sustainability. The foundational Food Choice Questionnaire developed by Steptoe, Pollard, and Wardle [70] demonstrated that food purchasing decisions are inherently multidimensional, reflecting the simultaneous consideration of health, convenience, price, mood, familiarity, and ethical concerns. More recent studies indicate that sustainability motives have become increasingly integrated into food choice processes, adding another layer of complexity to consumer decision-making [67]. Rather than maximizing a single attribute, consumers often attempt to balance multiple objectives simultaneously, making economic decision styles critical mechanisms through which these competing priorities are reconciled.

Recent research further suggests that food affordability, nutritional quality, and sustainability are interconnected but not always aligned. While healthier diets are generally associated with higher costs [65,66], food choices are influenced by a broader set of considerations, including taste preferences, convenience, habits, perceived value, and social norms [67,70]. Consequently, economic decision styles reflect not merely financial constraints but broader cognitive strategies through which consumers allocate limited resources across competing consumption goals. These trade-offs are particularly relevant in the context of sustainable diets, where consumers often seek to balance health, affordability, and environmental responsibility simultaneously [67].

The Generational Cohort Theory provides a useful framework for describing differences in economic decision-making across generational groups. Individuals exposed to similar social, economic, and cultural conditions during their formative years may develop partly shared values and behavioural tendencies [10]. Economic circumstances associated with different formative periods may therefore contribute to variation in approaches to food purchasing. Younger generations, particularly Generation Z, entered adulthood during periods characterized by economic uncertainty, inflation, rising housing costs, and labour market volatility. Regional studies suggest that such macro-economic experiences may foster stronger price consciousness, heightened sensitivity to promotions, and greater emphasis on maximizing value for money within this cohort [69,71].

At the same time, Generation Z consumers are frequently characterized by strong interest in sustainability, health, and ethical consumption [43]. This combination creates a unique decision-making environment in which economic constraints coexist with ambitious consumption goals. Empirical evidence suggests that Generation Z consumers often engage in complex trade-offs between affordability, healthfulness, sustainability, and convenience when selecting food products [69,71]. As a result, positive attitudes toward healthy and sustainable food may not always translate into corresponding purchasing behaviour, particularly when such products are perceived as more expensive. Economic decision styles may therefore help explain the frequently observed attitude–behaviour gap in food consumption.

Millennials exhibit a somewhat different economic profile. While remaining sensitive to food prices, this generation has historically appeared more willing to incur additional costs when products provide broader value through convenience, authenticity, health benefits, or sustainability attributes [67,72]. Research indicates that Millennials frequently evaluate food products using a multidimensional value framework that incorporates nutritional quality, convenience, lifestyle compatibility, and ethical considerations alongside economic concerns [43,72]. Consequently, economic decision-making among Millennials may be less focused purely on minimizing costs and more concerned with maximizing overall utility, though recent economic pressures have caused some segments of this cohort to reprioritize budget constraints.

In contrast, Generation X and Baby Boomers tend to demonstrate more established purchasing patterns characterized by stronger reliance on product quality, familiarity, trust, and previous purchasing experience [48,49]. Although economic considerations remain important for these cohorts, purchasing decisions are often embedded within broader assessments of reliability and perceived quality. Older consumers may therefore be less likely to engage in extensive price-comparison activities and more likely to rely on established purchasing habits when making food choices [69,71], though their increasing adaptation to digital retail platforms is gradually reshaping these traditional routines.

The growing importance of online food retailing introduces additional complexity into these generational differences. Online grocery shopping environments substantially increase price transparency by allowing consumers to compare alternatives, identify promotional offers, monitor price changes, and access personalized discounts with minimal effort [39]. While foundational research established how nutritional labels and sustainability claims shape food choices in physical retail settings [38], modern digital platforms reduce information-search costs further and actively facilitate more economically oriented decision-making processes. Furthermore, modern online food environments expose consumers to a dense array of dynamic ratings, reviews, and algorithmically targeted promotional messages that can fundamentally alter perceptions of value [37].

Research suggests that younger consumers are particularly adept at utilizing these digital tools to compare prices, evaluate value propositions, and optimize purchasing decisions [44,71]. Consequently, economic decision styles may exert a stronger influence on food purchasing behaviour in online environments than in conventional retail settings. However, the increased visibility of price information may also intensify conflicts between economic goals and other food choice motives, including healthy eating and environmental concern. Consumers may recognize the nutritional or environmental advantages of certain products while simultaneously selecting lower-priced alternatives due to explicit budgetary considerations.

The role of economic decision styles may become even more important in the context of increasing concerns regarding the affordability of healthy diets. As demonstrated by established nutritional economics literature, healthier dietary patterns are frequently associated with higher food expenditures, creating barriers to the adoption of recommended nutritional behaviours among price-sensitive consumers [65,66]. Rising food prices and broader economic uncertainty may further strengthen consumer sensitivity to price, potentially increasing the importance of economic decision styles in food purchasing decisions. Such developments are particularly relevant for younger generations, who often face greater financial constraints and may therefore be required to balance health and sustainability aspirations against strict budget limitations [69].

Despite growing interest in food choice motives, sustainable consumption, and online grocery shopping, relatively little attention has been devoted to understanding how economic decision-making interacts with healthy eating and environmental concern in shaping food purchasing decisions. Existing studies have typically examined these constructs separately, resulting in a fragmented understanding of their combined influence on consumer behaviour [67,69,73]. Furthermore, evidence regarding generational differences remains limited and sometimes inconsistent, particularly in digital food retail contexts. Given the increasing importance of online grocery shopping and the growing economic pressures facing consumers, further investigation of these relationships appears warranted.

Overall, the literature suggests that economic decision-making tendencies may be associated with online food purchasing and may vary across generational groups. Previous studies indicate that younger consumers may be more likely to engage in active price comparison, value optimization, and trade-offs between health, sustainability, and affordability, whereas older consumers may place relatively greater emphasis on quality, familiarity, and reliability. These patterns represent group-level tendencies rather than characteristics shared uniformly by all members of a given generation. Examining such variation may help clarify how economic considerations are associated with healthy eating, environmental concern, and online food purchase frequency.

### 2.5. Conceptual Model and Research Hypotheses

**Figure 1:**
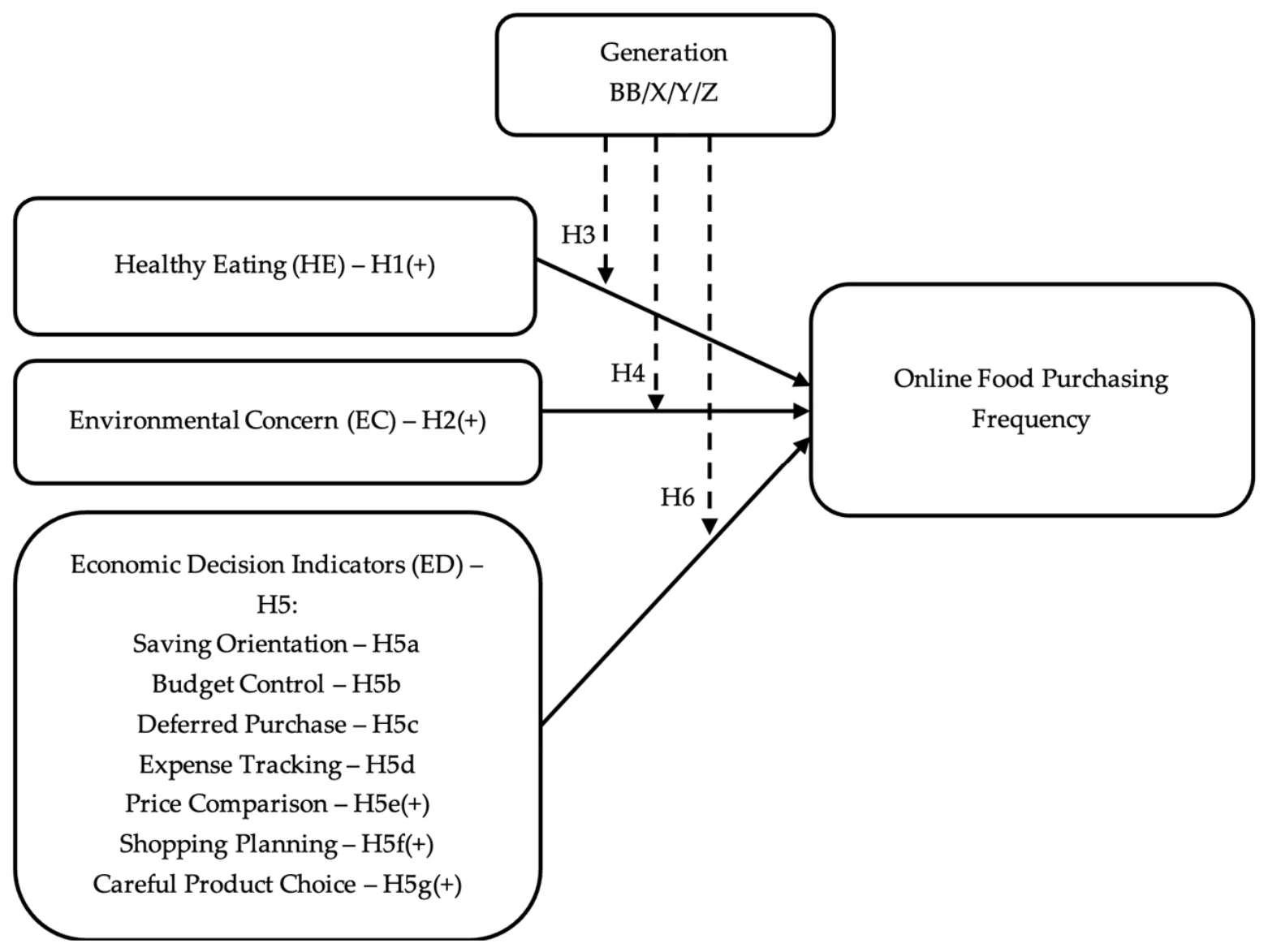
Conceptual research model. Note: solid arrows indicate hypothesized direct associations; dashed arrows indicate moderation hypotheses. Source: own preparation.

The conceptual model examines the associations of healthy eating (HE), environmental concern (EC), and each economic decision-making (ED) indicator with online food purchase frequency. Generational cohort is treated as an analytical grouping variable and potential moderator of these associations rather than as a self-sufficient causal mechanism. The outcome is operationalized in two complementary forms: a binary distinction between frequent and occasional online food purchasers and an eight-category ordinal measure of purchase frequency. The research model is presented in Figure 1.

The hypotheses reflect the theoretical arguments developed in the manuscript. Directional hypotheses are specified where the literature provides a sufficiently clear expectation. Directional hypotheses were specified for H1, H2, and H5e–H5g, whereas non-directional hypotheses were formulated for H5a–H5d and the moderation effects.

The hypotheses are as follows:

**H1.**  *Healthy eating is positively associated with the frequency of online food purchasing.*

**H2.**  *Environmental concern is positively associated with the frequency of online food purchasing.*

**H3.**  *Generational cohort moderates the relationship between healthy eating and online food purchasing frequency.*

**H4.**  *Generational cohort moderates the relationship between environmental concern and online food purchasing frequency.*

**H5.**  *Economic decision indicators are associated with online food purchasing frequency.*

**H5a.**  *Saving orientation is associated with online food purchasing frequency.*

**H5b.**  *Budget control is associated with online food purchasing frequency.*

**H5c.**  *Deferred purchase is associated with online food purchasing frequency.*

**H5d.**  *Expense tracking is associated with online food purchasing frequency.*

**H5e.**  *Price comparison is positively associated with online food purchasing frequency.*

**H5f.**  *Shopping planning is positively associated with online food purchasing frequency.*

**H5g.**  *Careful product choice is positively associated with online food purchasing frequency.*

**H6.**  *Generational cohort moderates the relationships between economic decision indicators and online food purchasing frequency.*

## 3. Materials and Methods

### 3.1. Questionnaire Development

The research instrument was a structured questionnaire developed by the research team for studies on the purchasing behaviour of e-consumers in the online environment. The instrument was progressively expanded and refined in successive waves of the research, drawing on the current literature on consumer behaviour and electronic commerce. These studies included “The Polish E-Consumer: Typology and Behaviour”, conducted in 2010 [74]; “The E-Consumer in Europe: A Comparative Analysis of Behaviour”, conducted in 2013 [75]; and “The Polish E-Consumer: A Decade of Change”, conducted in 2021 [3]. Given the broad thematic scope of the research project, the questionnaire covered numerous aspects of consumer activity in the digital environment. Therefore, brief forced-choice items were used instead of multi-item scales to limit respondent burden and maintain consistency with the previous research waves. However, for the purposes of the present article, only those questions directly related to the study objective were used, namely the analysis of intergenerational differences in online food purchasing, taking into account healthy eating, environmental concern, and economic decision-making.

In this study, online food purchasing referred to the purchase of food products from online stores and retailers. It did not include ordering prepared meals from restaurants or meal-delivery platforms. Online food purchasing was operationalized in two complementary ways. First, Frequent_Online_Food_Purchasing was used as a dichotomous outcome distinguishing frequent from occasional online food purchasers. Second, Online_Food_Purchase_Frequency was retained as an eight-category ordinal variable. The response categories were ordered from the highest to the lowest purchase frequency and coded from 1 to 8; consequently, higher numerical values represented progressively less frequent online food purchasing. Consequently, in the ordinal logistic regression models, an odds ratio below 1 indicated a greater likelihood of belonging to a category representing more frequent online purchasing, whereas an odds ratio above 1 indicated a greater likelihood of less frequent purchasing.

Healthy eating (HE) was assessed on the basis of e-consumers’ self-reported dietary patterns [3,74]. Environmental concern (EC) was understood as the declared tendency to engage in everyday pro-environmental behaviours. The inclusion of both variables was supported by numerous studies indicating that health-related and environmental motives are among the most important determinants of contemporary food choices [6,70,76,77]. Both HE and EC were included as binary explanatory variables. This binary operationalization reflected the structure of the original questionnaire and enabled comparisons between respondents declaring and not declaring each orientation; however, it did not capture the intensity or multidimensionality of these constructs. For HE, a value of 0 indicated no declared healthy eating, and a value of 1 indicated declared healthy eating. For EC, a value of 0 indicated no declared environmental concern and a value of 1 indicated declared environmental concern. These variables were examined in terms of their direct associations with online food purchase frequency and their interactions with generational group.

Economic decision-making (ED) described consumers’ preferred approaches to managing financial resources, planning purchases, comparing prices, controlling expenditure, and prioritizing quality. The design of this section of the questionnaire drew on the body of research on consumer decision-making, which was developed from the classical Consumer Styles Inventory framework [68,78]. Seven binary indicators were used to characterize respondents’ shopping tendencies: Saving_Orientation, Budget_Control, Deferred_Purchase, Expense_Tracking, Price_Comparison, Shopping_Planning, and Careful_Product_Choice. Each variable distinguished respondents who declared a given purchasing tendency from those who did not. This forced-choice format reflected the structure of the original questionnaire and captured the presence, but not the intensity, of each declared decision tendency. The variables were first examined separately and were subsequently entered simultaneously into a multivariable model to assess whether their associations with online food purchasing frequency remained after mutual adjustment.

The operationalization of the variables used in the analysis is presented in Table 1.

### 3.2. Data Collections

The study used a cross-sectional quantitative design. A quantitative research approach was adopted in this study using the Computer-Assisted Web Interviewing (CAWI) method, which enables the efficient collection of data from a large and diverse sample of respondents. This approach is widely employed in consumer behaviour research, including studies focusing on online shopping behaviour [79,80]. Before the main data collection phase, the research protocol received ethical approval from the Ethics Committee for Research Involving Human Participants at the University of Economics in Katowice (Approval No. 02/05/2025), confirming that the study complied with applicable ethical standards. Data were collected during the first half of 2025 using the SurveyMonkey platform. Respondents were recruited from a nationwide consumer research panel. Quota sampling was used to obtain 300 respondents from each of the four generational groups in the initial sample: Baby Boomers, Generation X, Generation Y, and Generation Z. The initial sample was therefore balanced across generational groups but was not intended to reflect the generational structure of the Polish population. Participation in the study was voluntary and anonymous. Before completing the questionnaire, all respondents were informed about the purpose of the study, its scientific nature, and the procedures for data processing. Informed consent was obtained from all participants, who were also informed of their right to withdraw from the study at any stage without providing a reason. The entire research procedure was conducted in accordance with the Declaration of Helsinki, applicable ethical standards, and the requirements of Regulation (EU) 2016/679 of the European Parliament and of the Council (General Data Protection Regulation, GDPR).

The survey invitation was distributed to approximately 10,000 members of a nationwide consumer panel. A total of 1371 individuals started the questionnaire and 1243 completed it, corresponding to a response rate of 12.43%. Following formal and substantive data-quality screening, 43 questionnaires were excluded because of missing responses to required items or unreliable response patterns. Consequently, 1200 questionnaires were retained for further analysis, including 300 respondents from each generational group. No post-stratification or statistical weighting was applied. Because the present study focused on purchase frequency among consumers who had previously purchased food online, 531 respondents who had never used this channel were excluded. The main analytical sample therefore comprised 669 respondents. One respondent had missing data for environmental concern; therefore, the regression models including HE and EC were estimated using 668 complete observations. Twenty-four respondents had missing data for at least one of the seven economic decision indicators. Consequently, complete data for all seven indicators were available for 645 respondents, who constituted the complete-case subsample for the mutually adjusted economic-indicator models.

### 3.3. Sample

Respondents were classified into four generational cohorts based on their year of birth: Baby Boomers (1946–1964), Generation X (1965–1979), Generation Y (1980–1994), and Generation Z (1995–2007). The largest group consisted of Generation Z followed by Generation Y, Baby Boomers, and Generation X. The sociodemographic structure of the sample varied across the analysed generations with respect to gender, household size, employment status, and self-assessed financial situation. The gender distribution was relatively balanced in all cohorts. Women slightly outnumbered men among Baby Boomers (54.5%) and Generation Z (50.5%), whereas men constituted a marginal majority in Generation X (53.7%) and Generation Y (53.0%).

Differences were also observed in household composition. Among Baby Boomers, two-person households were the most common (50.9%). In Generation X, four-person (30.9%) and three-person (30.1%) households predominated. Respondents from Generation Y most frequently lived in two-person (32.0%) and three-person (31.3%) households, whereas among Generation Z, two-person (21.5%) and three-person (30.05%) households were the most prevalent. Employment status differed considerably across the generations. The vast majority of respondents from Generation X (93.5%) and Generation Y (99.4%) were employed. In contrast, most Baby Boomers were not employed (64.2%), reflecting the age profile of this cohort. Among Generation Z, 72.0% of respondents were employed.

Across all generations, respondents generally assessed their financial situation as either good or sufficient. The highest proportion of respondents reporting a good financial situation was observed in Generation Y (61.3%), followed by Generation X (53.7%). In contrast, Baby Boomers most frequently described their financial situation as sufficient (47.3%). Regardless of generation, only a small proportion of respondents reported a poor or very poor financial situation. A detailed sociodemographic profile of the respondents is presented in Table 2.

### 3.4. Methods of Analysis

The analytical procedure consisted of descriptive, bivariate, and multivariable stages. All tests were two-sided, and the nominal significance level was set at α = 0.05. Odds ratios (ORs) were reported with 95% confidence intervals. Because the study involved multiple exploratory comparisons, the Benjamini–Hochberg procedure was used to control the false discovery rate. Healthy eating and environmental concern were initially examined separately within each generational cohort. For the dichotomous frequent purchasing outcome, differences between respondents with and without a given orientation were assessed using Pearson’s chi-square test. For the full eight-category purchasing frequency measure, the groups were compared using the Mann–Whitney U test. These analyses were treated as exploratory because several comparisons were conducted within each cohort.

Binary logistic regression was used for Frequent_Online_Food_Purchasing. Ordinal logistic regression with a logit link was used for Online_Food_Purchase_Frequency. The main-effects models included generation, HE, and EC. Moderation was tested by adding the HE × generation and EC × generation interaction blocks. Model fit was compared using likelihood-ratio tests based on changes in −2 log-likelihood. For the binary logistic models, log-likelihood, Akaike’s information criterion (AIC), and McFadden’s pseudo-R^2^ were additionally reported. A significant interaction block was interpreted as evidence that the association between the focal predictor and online food purchasing differed across generations. For significant interactions, generation-specific simple effects were estimated and expressed as regression coefficients, odds ratios, 95% confidence intervals, and *p* values.

The seven shopping-style indicators were examined using the same two outcome specifications. For each indicator, a separate binary logistic model and a separate ordinal logistic model were first estimated, controlling for generation. The direct association of the shopping-style variable with online food purchasing was then evaluated. Next, the corresponding shopping-style variable × generation interaction block was added and assessed using a likelihood-ratio test with three degrees of freedom. For statistically significant interactions, simple effects were calculated separately for BB, Gen. X, Y, and Z. An interaction with opposite directions across generational groups was identified when the direction of the association differed between at least two groups. Finally, all seven shopping-style variables were entered simultaneously into multivariable binary and ordinal logistic regression models. These models were used to assess whether direct and moderating effects remained after mutual adjustment for the remaining shopping-style characteristics.

Results from separate predictor models were interpreted together with the multivariable models and the Benjamini–Hochberg-adjusted *p* values. Effects that were statistically significant only in isolated models but not after simultaneous adjustment or correction for multiple testing were classified as exploratory rather than robust. Conclusions regarding moderation were based primarily on the joint tests of the interaction blocks and on the consistency of findings across the binary and ordinal outcome specifications.

The proportional-odds assumption was assessed using a Brant-type joint Wald test. The assumption was not fully satisfied, as Wald χ^2^(54) = 73.62, *p* = 0.039; therefore, the ordinal models were interpreted as sensitivity analyses.

Because the study was cross-sectional, regression coefficients were interpreted as associations rather than causal effects. Birth cohort was treated as an analytical grouping variable and moderator; the models cannot distinguish cohort effects from age and life-stage differences.

## 4. Results

### 4.1. Within-Generation Analyses

#### 4.1.1. Healthy Eating

Within-generation comparisons provided no evidence that declared healthy eating was associated with online food purchasing frequency. The proportion of frequent purchasers was numerically higher among respondents declaring healthy eating in the Baby Boomer, Generation X, and Generation Z cohorts, whereas the opposite pattern was observed in Generation Y. However, none of the binary comparisons was statistically significant, and all confidence intervals for the odds ratios included 1. The Mann–Whitney U tests based on the full eight-category frequency measure likewise showed no significant differences. All results remained non-significant after the Benjamini–Hochberg correction, indicating that healthy eating was not associated with purchasing frequency within any of the four generations (Table 3).

#### 4.1.2. Environmental Concern

The associations between environmental concern and online food purchasing showed a different pattern across generations. In the binary comparisons, environmental concern was associated with a higher proportion of frequent purchasers among Baby Boomers, Generation X, and Generation Y, whereas the direction was reversed in Generation Z. The uncorrected binary comparisons reached nominal significance among Baby Boomers and Generation Z, but neither remained significant after the Benjamini–Hochberg correction (both *p*BH = 0.077). The ordinal comparisons revealed a clearer generational contrast. Among Baby Boomers, respondents declaring environmental concern purchased food online more frequently than those without such concern (medians: 5 vs. 6, *p*BH = 0.033). In Generation Z, environmental concern was associated with less frequent purchasing (medians: 3 vs. 1, *p*BH = 0.033). No significant ordinal differences were found in Generations X or Y (Table 4).

Because multiple bivariate analyses were conducted, the Benjamini–Hochberg correction was applied. These analyses should therefore be regarded as exploratory, whereas the formal tests of the interaction blocks presented below provide the basis for evaluating moderation.

### 4.2. Binary Logistic Regression for Frequent Online Food Purchasing

Binary logistic regression models were subsequently estimated to formally test whether the associations of healthy eating and environmental concern with frequent online food purchasing differed across generations. Adding the HE × generation interaction did not significantly improve the main-effects model in either the unadjusted specification, Δχ^2^(3) = 3.51, *p* = 0.319, or the sociodemographic-adjusted specification, Δχ^2^(3) = 3.37, *p* = 0.338. In contrast, the EC × generation interaction significantly improved model fit before adjustment, Δχ^2^(3) = 12.80, *p* = 0.005, and after adjustment for gender, employment status, household size, and self-assessed financial situation, Δχ^2^(3) = 12.13, *p* = 0.007. The EC-interaction models also had the lowest AIC values within both specifications. The full models containing both interaction blocks improved fit relative to the corresponding main-effects models, but adding the HE interaction provided no additional substantive improvement (Table 5). Detailed results of the full sociodemographic-adjusted models are presented in Appendix A (Table A1 and Table A2).

Generation-specific estimates confirmed the absence of a significant association between healthy eating and frequent online food purchasing in any cohort. The adjusted odds ratios for HE ranged from 0.61 in Generation Y to 1.35 in Generation X, and all confidence intervals included 1. Environmental concern showed a contrasting pattern across generations. Its association with frequent online purchasing was positive among Baby Boomers and Generations X and Y, with adjusted odds ratios ranging from 1.89 to 3.57, but negative in Generation Z, OR = 0.47. The unadjusted EC association was significant in Generation Z, but none of the generation-specific EC estimates remained individually significant after sociodemographic adjustment. The significant joint EC × generation test reported in Table 5 therefore reflects heterogeneity in the direction of the association across cohorts rather than a uniformly significant association within each generation (Table 6).

### 4.3. Ordinal Logistic Regression for the Full Purchasing-Frequency Scale

The ordinal logistic regression models produced a pattern consistent with the binary analyses. Adding the HE × generation interaction did not improve model fit in either the unadjusted model, Δχ^2^(3) = 2.78, *p* = 0.427, or the adjusted model, Δχ^2^(3) = 2.91, *p* = 0.406. By contrast, the EC × generation interaction significantly improved both the unadjusted model, Δχ^2^(3) = 13.58, *p* = 0.004, and the adjusted model, Δχ^2^(3) = 12.77, *p* = 0.005. The full interaction models were also significantly better than the corresponding main-effects models. As in the binary analysis, the EC-interaction specification had a lower AIC than the HE-interaction and full models. Because the proportional-odds assumption was not fully satisfied, the ordinal results were interpreted as sensitivity analyses supporting the findings obtained from the binary models (Table 7).

The generation-specific ordinal estimates showed no significant association between healthy eating and purchasing frequency in any cohort. Environmental concern, however, was significantly associated with purchasing frequency among Baby Boomers and Generation Z but in opposite directions. After sociodemographic adjustment, environmental concern was associated with a greater probability of more frequent purchasing among Baby Boomers, OR = 0.45, 95% CI [0.21, 0.96], *p* = 0.039. In Generation Z, it was associated with a greater probability of less frequent purchasing, OR = 2.54, 95% CI [1.23, 5.27], *p* = 0.012. The estimates for Generations X and Y were in the same direction as the Baby Boomer estimate but were not statistically significant. These findings indicate a qualitative EC × generation interaction and corroborate the generational heterogeneity observed in the binary analysis (Table 8).

### 4.4. Direct Associations of Shopping-Style Variables

In separate models, nominally significant direct associations were observed for Price_Comparison and Careful_Product_Choice. Price comparison increased the odds of frequent online food purchasing by approximately 69%:

OR = 1.69, 95% CI [1.09, 2.61], *p* = 0.019.

This result was confirmed in the ordinal model:

OR = 0.63, 95% CI [0.44, 0.91], *p* = 0.013.

Because lower values indicated more frequent purchasing, the odds ratio below 1 in the ordinal model showed that respondents who compared prices were more likely to purchase food online more frequently than respondents who did not compare prices.

A statistically significant association was also found for Careful_Product_Choice. In the binary logistic model, respondents who carefully selected products had approximately 53% higher odds of frequent online purchasing:

OR = 1.53, 95% CI [1.04, 2.24], *p* = 0.033.

The ordinal model yielded a consistent result:

OR = 0.70, 95% CI [0.51, 0.97], *p* = 0.030.

No statistically significant direct associations were observed for Saving_Orientation, Budget_Control, Deferred_Purchase, Expense_Tracking, or Shopping_Planning. For these predictors, *p* values exceeded 0.05 in both the binary and ordinal logistic regression models. Detailed results for the economic decision indicators in separate and fully adjusted models are presented in Appendix A (Table A3).

### 4.5. Moderating Role of Generation

The moderating role of the generational cohort was then examined. A statistically significant and consistent interaction across both operationalizations of purchasing frequency was obtained for Saving_Orientation. In the binary logistic model, adding the Saving_Orientation × Generation interaction significantly improved model fit:

Δχ^2^(3) = 10.48, *p* = 0.015.

A corresponding effect was found in the ordinal model:

Δχ^2^(3) = 11.41, *p* = 0.010.

Simple-effect analyses showed that the direction of the association between saving orientation and online food purchasing frequency differed across generations. In the ordinal model, Saving_Orientation was associated with more frequent online food purchasing in Generation X:

OR = 0.43, 95% CI [0.21, 0.90], *p* = 0.025.

In Generation Z, the association was reversed. Respondents with a stronger saving orientation were more likely to belong to categories representing less frequent online food purchasing:

OR = 2.48, 95% CI [1.03, 5.98], *p* = 0.043.

The simple effects were not statistically significant among Baby Boomers or Generation Y. The interaction was therefore qualitative, because the direction of the relationship changed across generational cohorts: saving orientation was associated with more frequent online purchasing in Generation X but with less frequent purchasing in Generation Z.

For Shopping_Planning, the interaction with generation was statistically significant only in the binary logistic model:

Δχ^2^(3) = 8.14, *p* = 0.043.

The simple effect was significant only in Generation Y. Within this cohort, respondents who planned their purchases had approximately twice the odds of frequent online food purchasing:

OR = 2.04, 95% CI [1.13, 3.71], *p* = 0.019.

However, the Shopping_Planning × Generation interaction did not reach statistical significance in the ordinal model:

Δχ^2^(3) = 6.96, *p* = 0.073.

The moderating effect of shopping planning was therefore not consistently confirmed across the two model specifications.

No statistically significant interactions with generation were found for Budget_Control, Deferred_Purchase, Expense_Tracking, Price_Comparison, or Careful_Product_Choice. Thus, the associations between these shopping-style characteristics and online food purchasing frequency did not differ significantly across generations. Detailed results of the generation-moderation tests for the economic decision indicators are presented in Appendix A (Table A4).

### 4.6. Multivariable Model

In the final multivariable model, all seven shopping-style characteristics were entered simultaneously. After mutual adjustment of the predictors, none of the direct associations reached the conventional significance threshold of *p* < 0.05. Price_Comparison continued to show the strongest association, although its effect in the binary logistic model was only close to statistical significance:

OR = 1.50, *p* = 0.092.

In the ordinal model, the result for Price_Comparison was not at the conventional threshold of statistical significance:

OR = 0.70, *p* = 0.078.

No interactions with generation remained statistically significant after the Benjamini–Hochberg correction. The Saving_Orientation × generation interaction was nominally significant in the ordinal model (*p* = 0.029) but not after the BH correction (*p*BH = 0.206) and Shopping_Planning × generation in the binary logistic model (*p* = 0.080; *p*BH = 0.253).

### 4.7. Adjustment for Multiple Testing

The economic-indicator results were finally evaluated after correction for multiple testing and simultaneous adjustment for the remaining shopping-style characteristics. Price comparison and careful product choice were nominally associated with more frequent purchasing in the separate binary and ordinal models. Saving orientation showed nominal interactions with generation in both outcome specifications, while shopping planning showed a nominal interaction only in the binary model. However, none of these separate-model findings remained significant after the Benjamini–Hochberg correction. In the fully adjusted models, no direct association remained statistically significant. The Saving_Orientation × generation interaction was nominally significant in the adjusted ordinal model (*p* = 0.029) but not after correction for multiple testing (*p*BH = 0.206). Consequently, none of the economic associations met the criteria for a robust result after both multivariable adjustment and false-discovery-rate control (Table 9).

## 5. Discussion

The purpose of this study was to examine whether healthy eating, environmental concern, and economic decision indicators were associated with online food purchase frequency and whether these associations differed across generational groups. The findings suggest that the associations were not uniform across the examined groups. The clearest difference concerned the association between environmental concern and purchase frequency, whereas the results for the economic decision indicators were predominantly exploratory. These patterns are consistent with Generational Cohort Theory, although the cross-sectional design does not allow cohort-related differences to be separated from age, life stage, household circumstances, or familiarity with digital technologies [10]. Healthy eating was not significantly associated with online food purchasing frequency in either the binary or ordinal models. This association also did not differ significantly across generational groups. Consequently, H1 and H3 were not supported. This finding does not imply that healthy eating orientation is irrelevant to food choice. Previous studies have associated health consciousness with attitudes toward food, attention to nutritional attributes, label use, and intentions to purchase healthier products [9,13,16,18,22,23,24,25,26,27,28,29]. The present results indicate, however, that declaring a healthy eating orientation was not associated with how frequently respondents used the online channel to purchase food.

One possible explanation is that healthy eating may be more relevant to product selection rather than channel-use intensity. Consumers may apply health-related criteria in both online and physical stores, while their choice of channel may also depend on convenience, delivery options, platform usability, retailer trust, product availability, and established shopping routines [37,54,63,64]. Online purchasing frequency should therefore not be treated as a direct behavioural equivalent of healthy food purchasing. A health-oriented consumer may carefully examine nutritional information online but purchase food digitally only occasionally, whereas a consumer without a declared healthy eating orientation may purchase online frequently because of convenience or time savings.

The absence of a significant association may also reflect the complexity of digital food environments. Online platforms provide extensive nutritional information and facilitate product comparison, but they simultaneously expose consumers to substantial volumes of competing information, promotional content, ratings, and claims [37,56,81]. Excessive information may reduce consumers’ ability to consistently incorporate health-related considerations into their decisions [59,60,61,62]. The frequent use of heuristics and familiar-product choices may further weaken the behavioural expression of health orientation [66]. The present findings are therefore compatible with the literature suggesting that health consciousness increases sensitivity to nutritional attributes without necessarily producing a simple or uniform change in purchasing behaviour [25,28,29].

Environmental concern produced a substantially different pattern. Its main effect was not uniform across the full sample, but its interaction with generation was statistically significant in both outcome specifications. H2 was therefore not supported as a general direct-effect hypothesis, whereas H4 was supported. Among Baby Boomers, environmental concern was associated with more frequent online food purchasing, particularly in the ordinal model. Among Generation Z, the relationship was reversed: environmentally concerned respondents purchased food online less frequently than those who did not declare such concern. The effects observed for Generations X and Y were not statistically significant.

The positive association identified among Baby Boomers may indicate that environmentally concerned members of this cohort use online channels selectively as instruments for obtaining information and accessing products that meet broader quality, health, local-origin, or sustainability criteria. Previous research suggests that older consumers tend to integrate environmental benefits into assessments of product utility, freshness, quality, trust, and health rather than treating environmental attributes as isolated purchase motives [48,49,50]. Digital retail environments can support such evaluations by providing information on product origin, certification, sourcing, and packaging [37,39,40]. For environmentally concerned Baby Boomers who have already adopted online food purchasing, digital channels may therefore facilitate more deliberate and information-based choices.

The reverse association found in Generation Z is theoretically important because younger consumers are often described as environmentally aware, digitally competent, and receptive to sustainable consumption [41,42,43,44]. The result does not necessarily contradict this literature. Instead, it may reflect the frequently observed gap between environmental concern and actual behaviour [52,53]. Environmentally concerned Generation Z consumers may perceive some characteristics of online food retailing, such as additional packaging, transport, delivery-related emissions, minimum-order requirements, or reduced access to local products, as inconsistent with their sustainability objectives. They may therefore restrict online food purchasing despite being highly competent users of digital technologies.

The Generation Z finding may also reflect economic trade-offs. Younger consumers often combine strong health and sustainability aspirations with limited financial resources, labour-market uncertainty, and high price sensitivity [42,69,71]. Environmentally preferable food options may involve price premiums, while online purchasing may entail delivery charges or encourage larger basket sizes. Consequently, environmentally concerned young consumers may reduce purchasing frequency or use physical stores when these channels provide greater control over product choice, packaging, and expenditure. This interpretation is consistent with evidence that sustainable food decisions are shaped simultaneously by environmental values, affordability, convenience, and perceived personal benefits [35,36,50,67].

The opposite directions observed for Baby Boomers and Generation Z also suggest why environmental concern should not be interpreted as a generationally invariant predictor. Digital competency alone may not explain whether environmental attitudes translate into online purchasing. The observed pattern may depend on how members of each cohort define sustainable purchasing, evaluate online delivery, integrate environmental claims with product quality and price, and perceive the credibility of information available online [36,44,51]. The findings therefore extend previous research by suggesting that the same declared environmental orientation may be associated with more frequent online purchasing in one cohort and less frequent purchasing in another. The analyses of economic decision indicators provided predominantly exploratory evidence for H5 and its sub-hypotheses. In the separate predictor models, price comparison and careful product choice were associated with more frequent online food purchasing in both binary and ordinal specifications. These findings were directionally consistent with H5e and H5g. No statistically significant direct effects were found for saving orientation, budget control, deferred purchase, expense tracking, or shopping planning; therefore, H5a–H5d and H5f were not supported as general direct-effect hypotheses.

The association between price comparison and more frequent online food purchasing is consistent with the structure of digital retail environments. Online channels reduce search costs, increase price transparency, and allow consumers to compare products, retailers, promotions, and delivery conditions with relatively little effort [39]. Consumers characterized by price-comparison tendencies may consequently derive greater utility from online shopping than consumers who do not actively compare alternatives. This interpretation corresponds with earlier findings that price, convenience, platform usability, product assortment, and access to information jointly influence online grocery channel selection [37,63,64].

Careful product choice was also associated with more frequent online purchasing in separate models. This result suggests that online food retailing may be attractive not only to convenience-oriented consumers but also to deliberative consumers who require time and information before making a decision. Digital platforms allow repeated examination of product descriptions, ingredient lists, nutritional information, labels, reviews, and alternative offers without the time pressure typically associated with physical stores [13,28,37]. Such an environment may support consumers who carefully evaluate products and take longer to choose among alternatives.

Nevertheless, the associations of price comparison and careful product choice weakened after all seven decision-making indicators were entered simultaneously. Neither effect was consistently robust after the Benjamini–Hochberg correction, although price comparison remained the strongest direct association and reached the conventional significance threshold in the ordinal multivariable model. These results suggest that the economic decision-making variables may not represent fully independent behavioural tendencies. Price comparison, careful product selection, planning, saving, and budget control may represent overlapping elements of a broader deliberative purchasing orientation. The classical literature on consumer decision styles similarly treats purchasing behaviour as multidimensional and recognizes that several decision tendencies may coexist within the same consumer [68,78].

Generation significantly moderated the association between saving orientation and online food purchasing in the separate binary and ordinal models. The association differed in direction across generational groups: saving orientation was associated with more frequent purchasing in Generation X but with less frequent purchasing in Generation Z. This pattern provided provisional support for H6. However, the interaction was not statistically significant after multiple-testing correction or simultaneous adjustment and must therefore be interpreted as exploratory.

For Generation X, online food purchasing may function as a tool for economical resource management. Members of this cohort may use digital price transparency, promotional offers, shopping-list functions, and reduced travel requirements to control expenditure and obtain better value [39,63]. Saving orientation may therefore be associated with more frequent use of online food channels when these channels are perceived as efficient instruments for household-budget management.

In Generation Z, by contrast, saving orientation was associated with less frequent online purchasing. Younger consumers may regard delivery fees, minimum basket values, impulse-stimulating digital interfaces, and limited control over final product quality as potential sources of unnecessary expenditure. A saving-oriented Generation Z consumer may therefore restrict online food purchasing or use it only selectively. This explanation is consistent with evidence that younger consumers face strong economic pressures and negotiate complex trade-offs between affordability, convenience, sustainability, and health [42,69,71].

Shopping planning showed a significant interaction with generation only in the binary model. Within Generation Y, planned purchasing was associated with approximately twice the odds of frequent online food purchasing. Online grocery platforms may be particularly compatible with planned shopping because they allow consumers to create lists, repeat earlier orders, monitor basket values, and complete routine household purchases efficiently. However, the interaction was not significant in the ordinal model and did not remain significant in the multivariable analysis. The evidence was therefore insufficient to confirm a stable moderating effect.

No generational moderation was found for budget control, deferred purchase, expense tracking, price comparison, or careful product choice. These results indicate that some economic decision tendencies may operate similarly across age cohorts, even when the prevalence of online purchasing differs among generations. In particular, the usefulness of online channels for price comparison and detailed product evaluation may not be confined to younger digital consumers. The increasing adoption of online retailing among older cohorts may be contributing to a convergence in some digitally supported purchasing practices [3,48,49].

Taken together, the findings provide only limited support for a simple generational profiling approach based on average cohort characteristics. Generation did not consistently moderate the examined associations and should not be treated as a deterministic explanation of consumer behaviour. Its clearest statistical role was that of a contextual moderator for selected orientations, especially environmental concern. This conclusion is consistent with studies showing both divergence and convergence in food-related behaviour across generations [69,71,73]. Generational groups may differ in values, economic experiences, and technological socialization, but substantial heterogeneity remains within each group.

The results also reinforce the multidimensional character of food choice. Consumers do not maximize a single objective but balance health, environmental responsibility, price, convenience, familiarity, quality, and accessibility [36,67,70]. Online food purchasing further intensifies these trade-offs by making information and price comparisons easier while introducing concerns relating to delivery, packaging, the credibility of claims, and information overload [37,51,56,59]. Consequently, none of the examined orientations or individual economic decision tendencies were sufficient to explain online food purchase frequency independently.

The low pseudo-R^2^ of the binary regression model additionally indicates that much of the variation in online food purchasing remains unexplained by the present model. Potentially relevant factors not included in the analysis include perceived convenience, trust in online retailers, delivery availability, previous online shopping experience, digital skills, household structure, place of residence, platform usability, perceived product quality, and situational constraints [37,54,63,64]. The model should therefore be interpreted as an exploratory examination of selected motivational and decision-related associations rather than as an exhaustive account of online food purchase frequency.

## 6. Practical Implications

The findings suggest that online food retailers, food producers, platform designers, and organizations responsible for consumer and sustainability communication should be cautious when assuming that health, environmental, and economic orientations are associated with identical online purchasing patterns across generational groups. Similar declarations may be associated with different patterns of online food purchase frequency. Generational group should therefore not be used as the sole basis for personalization or communication strategies. The absence of a significant association between healthy eating and purchase frequency suggests that health-related orientation alone should not be assumed to increase the use of online food channels. Features such as standardized nutritional information, ingredient lists, allergen information, and dietary filters may support health-oriented product selection; however, their influence on online food purchase frequency was not examined in this study and should be evaluated in future research [13,28,37].

The generation-dependent association involving environmental concern suggests that uniform sustainability communication may not be equally relevant across generational groups. Among Baby Boomers, the positive association between declared environmental concern and purchase frequency may justify further research into whether credible information on product origin, freshness, certification, or other functional benefits contributes to the use of online food channels [48,49,50]. These factors were not measured in the present study and should therefore be treated as directions for future research rather than evidence-based recommendations. Among Generation Z, the negative association between environmental concern and purchase frequency raises questions about whether some environmentally concerned consumers perceive online fulfilment as inconsistent with their sustainability objectives. Future research should examine the possible roles of packaging, delivery-related emissions, delivery options, and the credibility of environmental information in this relationship [42,43,44,45,46]. Because these factors were not measured in the present study, no specific platform or communication interventions can be recommended on the basis of the current findings. The exploratory differences in the association between saving orientation and purchase frequency suggest that economic considerations may not operate uniformly across generational groups. Future studies could examine whether delivery charges, minimum-order thresholds, price-comparison tools, and budget-management functions contribute to these differences. Because the interaction did not remain significant after multiple-testing correction or simultaneous adjustment, it does not provide a sufficient basis for generation-specific recommendations. The Generation Y result for shopping planning was not robust across model specifications. The possible relevance of saved baskets, recurring lists, reminders, previous-order replication, and delivery-scheduling functions should therefore be examined in future studies rather than treated as a direct implication of the present findings. The findings should not be interpreted as justification for rigid age-based segmentation. The statistical results indicate substantial within-generation heterogeneity and limited robustness of several cohort-specific associations. Generational group should therefore not be used as the sole basis for personalization or communication strategies. Future research should examine whether combining generational information with behavioural data, declared preferences, purchase frequency, and platform-use patterns provides a more informative basis for consumer segmentation.

## 7. Limitations and Future Research

This study has several limitations. Its cross-sectional design does not allow causal inference. The analyses identify associations between declared orientations, decision tendencies, generation group, and online food purchase frequency, but they do not establish whether these characteristics cause consumers to purchase food online more or less frequently. Longitudinal studies should examine whether changes in environmental concern, economic circumstances, or digital experience are followed by changes in online food purchasing behaviour.

All variables were based on self-reported declarations. Purchasing frequency, healthy eating, environmental concern, and economic decision tendencies may therefore be affected by recall errors and social-desirability bias. This issue is particularly relevant for health and environmental variables because respondents may overstate socially approved orientations. Future studies should combine survey data with transaction records, platform-use data, actual basket composition, or experimental purchase tasks.

HE and EC were operationalized as binary indicators. This approach provided a clear distinction between respondents declaring and not declaring each orientation, but it reduced the variability and multidimensionality of the constructs. Future studies should use validated multi-item scales to distinguish these dimensions and assess their reliability and validity. The simplified binary coding may also have resulted in measurement error and loss of information, potentially reducing statistical power and attenuating or destabilizing the estimated associations.

The economic decision-making indicators were also binary forced-choice measures. Although they captured practically relevant contrasts in purchasing tendencies, they did not measure the intensity of each style. Future research should employ multi-item scales derived from the Consumer Styles Inventory or related frameworks and assess their dimensional structure before estimating direct and moderating effects.

The study examined online purchasing frequency rather than the nutritional or environmental characteristics of the food purchased. Frequent online purchasing should not be interpreted as healthier or more sustainable purchasing. Future research should distinguish channel usage from product selection and examine whether health and environmental orientations predict the proportion of healthy, organic, local, minimally packaged, or sustainably certified products in online baskets.

The analytical sample consisted only of respondents who had purchased food online. The study therefore explains variation in purchasing frequency among online food purchasers but does not explain initial adoption or non-adoption. Comparisons including consumers who have never purchased food online would allow researchers to distinguish factors associated with channel adoption from those associated with continued or frequent use.

The sample was drawn from Poland, and the findings may reflect the specific development of Polish e-commerce, food retailing, delivery infrastructure, and consumer purchasing power. The meaning of generational categories may also differ across countries because cohorts experience different political, economic, and technological conditions. Cross-national research should test whether the observed environmental-concern interaction and exploratory saving-orientation interaction occur in markets with different levels of online grocery penetration and environmental awareness.

Generational cohort was defined according to birth-year boundaries. Such classifications simplify continuous age differences and may combine individuals with diverse life experiences. Age, life-cycle position, cohort effects, employment status, household composition, and period effects cannot be fully separated in a single cross-sectional study. Future research should use age–period–cohort designs or longitudinal data where possible and should compare generational models with models based on continuous age and life-stage variables.

Although the adjusted models included gender, employment status, household size, and self-assessed financial situation, residual confounding by education, place of residence, other household characteristics, and additional age- or life-stage-related factors cannot be excluded. Future studies should include a broader range of sociodemographic covariates to assess whether the observed generation-dependent associations remain after more comprehensive adjustment. The regression models explained a limited proportion of variation in purchasing frequency. Several potentially important predictors were not included, such as perceived convenience, trust, perceived risk, digital competence, retailer availability, delivery costs, urban or rural residence, household responsibilities, product assortment, previous experience, and platform usability. Including these variables could provide a more comprehensive explanation of online food purchasing.

Several findings were statistically significant only in separate predictor models and did not remain significant after correction for multiple testing or simultaneous adjustment. Price comparison, careful product choice, saving orientation, and shopping planning should therefore be regarded as exploratory findings requiring replication.

## 8. Conclusions

This study examined whether healthy eating, environmental concern, and economic decision indicators were associated with online food purchasing frequency and whether these associations differed among Baby Boomers, Generation X, Generation Y, and Generation Z. In this sample of Polish online food purchasers, the associations between the declared orientations and purchase frequency were not fully uniform across generational groups.

Healthy eating was not associated with purchasing frequency nor interacted significantly with generation group. Thus, declaring a healthy eating orientation did not distinguish frequent from occasional online food purchasers and was not associated with the full distribution of purchasing frequency. Health-related motivations may be more relevant to the types of products selected than to the frequency with which the online channel is used.

Environmental concern showed the clearest generation-dependent association with online food purchase frequency. It was associated with more frequent online purchasing among Baby Boomers but with less frequent purchasing among Generation Z. This difference suggests that the association between declared environmental concern and channel-use frequency may not be uniform across generational groups. Older and younger consumers may evaluate the environmental benefits and costs of online food retailing differently.

Price comparison and careful product choice were associated with more frequent online purchasing in separate models, suggesting that digital food retailing may be particularly relevant to economically oriented and deliberative purchasing. However, these associations weakened after adjustment for other decision indicators and correction for multiple testing. Saving orientation also showed opposite associations in Generations X and Z, but this interaction was exploratory rather than robust.

The principal conclusion is that generation group may be more informative as a moderator of selected behavioural associations than as a universal explanatory category. The analysis of online food purchasing should therefore consider not only consumers’ declared orientations but also the possibility that their associations with channel-use frequency may differ across generational groups.

## Figures and Tables

**Table 1:** Operationalization of variables used in the analyses.

Variable	Role	Operationalization/Coding
Generation	Grouping variable/moderator	Categorical: BB, Generation X, Generation Y, Generation Z BB used as reference category
Frequent_Online_Food_Purchasing	Dependent variable	Binary measure: 1 = frequent online purchases, defined as purchases made at least several times a month 0 = occasional online purchases, defined as purchases made less than once a month
Online_Food_Purchase_Frequency	Dependent variable	Eight-category ordinal measure: 1 = several times a week 2 = once a week 3 = several times a month 4 = once a month 5 = several times a year 6 = once a year 7 = less than once a year 8 = only once
HE	Predictor	0 = I eat unhealthy and irregularly 1 = I eat healthily and systematically
EC	Predictor	0 = I don’t care about the environment 1 = I care about the environment
Saving_Orientation	Economic decision tendencies predictor	0 = I buy the best products regardless of price 1 = I buy what I need while trying to manage my money economically
Budget_Control	Economic decision tendencies predictor	0 = If I want something, I buy it without considering whether I can afford it at the time 1 = Before buying something, I carefully check whether I can afford it
Deferred_Purchase	Economic decision tendencies predictor	0 = I often take out a loan or use credit to buy something more expensive 1 = Before buying something more expensive, I save the necessary money
Expense_Tracking	Economic decision tendencies predictor	0 = Keeping records of expenses is a waste of time and serves no useful purpose 1 = I record my expenses because it helps me manage my money.
Price_Comparison	Economic decision tendencies predictor	0 = I do not compare prices across different stores 1 = I check prices in different stores and try to buy at the lowest possible price
Shopping_Planning	Economic decision tendencies predictor	0 = I decide what to buy only when I am in the store 1 = I go shopping with a detailed list and buy only the items included on it
Careful_Product_Choice	Economic decision tendencies predictor	0 = I do not give much thought to product selection 1 = I carefully examine different products and take a long time to make my choice

Source: own preparation.

**Table 2:** Characteristics of the analytical sample by generation (%).

Item		BB (*n* = 165)	X (*n* = 123)	Y (*n* = 181)	Z (*n* = 200)
Gender	Women	90 (54.5)	57 (46.3)	85 (47.0)	101 (50.5)
	Men	75 (45.5)	66 (53.7)	96 (53.0)	99 (49.5)
Number of household members	1 person	32 (19.4)	11 (8.9)	24 (13.3)	34 (17.0)
	2 persons	84 (50.9)	20 (16.3)	58 (32.0)	43 (21.5)
	3 persons	30 (18.2)	37 (30.1)	57 (31.5)	60 (30.0)
	4 persons	16 (9.7)	38 (30.9)	34 (18.8)	44 (22.0)
	5 persons and more	3 (1.8)	17 (13.8)	8 (4.4)	19 (9.5)
Employment status	Currently employed	59 (35.8)	115 (93.5)	180 (99.4)	144 (72.0)
	Not currently employed	106 (64.2)	8 (6.5)	1 (0.6)	56 (28.0)
Self-assessment of the material situation	Very good	12 (7.3)	16 (13.0)	34 (18.8)	23 (11.5)
	Good	66 (40.0)	66 (53.7)	111 (61.3)	86 (43.0)
	Sufficient	78 (47.3)	34 (27.6)	33 (18.2)	76 (38.0)
	Bad	8 (4.8)	7 (5.7)	2 (1.1)	12 (6.0)
	Very bad	1 (0.6)	0 (0.0)	1 (0.6)	3 (1.5)

Source: own preparation.

**Table 3:** Online food purchasing by healthy eating across generations.

Generation	Frequent: HE = 0	Frequent: HE = 1	Pearson chi2	*p*	*p*BH	OR [95% CI]	Median: HE = 0/HE = 1	*p* (U)	*p*BH (U)
BB	28.3%	31.4%	0.17	0.677	0.677	1.16 [0.58, 2.33]	5/5	0.624	0.624
X	30.2%	41.4%	1.64	0.200	0.433	1.64 [0.77, 3.48]	5/5	0.179	0.597
Y	52.1%	42.7%	1.53	0.216	0.433	0.69 [0.38, 1.25]	3/5	0.377	0.597
Z	51.5%	55.7%	0.36	0.550	0.677	1.18 [0.68, 2.07]	3/2	0.447	0.597

Note. HE = 0 indicates no declared healthy eating orientation; HE = 1 indicates declared healthy eating orientation. OR > 1 indicates higher odds of frequent online purchasing in the HE = 1 group. Medians refer to the eight-category outcome, where lower values indicate more frequent purchasing. BH correction was applied separately to the four binary and four ordinal comparisons. HE = 0/HE = 1 group sizes: BB: 60/105; X: 53/70; Y: 71/110; Z: 103/97. Source: own preparation.

**Table 4:** Online food purchasing by environmental concern across generations.

Generation	Frequent: EC = 0	Frequent: EC = 1	Pearson chi2	*p*	*p*BH	OR [95% CI]	Median: EC = 0/EC = 1	*p* (U)	*p*BH (U)
BB	12.5%	33.6%	4.29	0.038	0.077	3.54 [1.00, 12.47]	6/5	0.015	0.033
X	16.7%	40.0%	3.61	0.058	0.077	3.33 [0.91, 12.23]	5/4	0.082	0.109
Y	36.0%	48.1%	1.26	0.261	0.261	1.65 [0.69, 3.95]	5/4	0.364	0.364
Z	68.6%	50.3%	3.87	0.049	0.077	0.46 [0.21, 1.01]	1/3	0.016	0.033

Note. EC = 0 indicates no declared environmental concern; EC = 1 indicates declared environmental concern. OR > 1 indicates higher odds of frequent online purchasing in the EC = 1 group. Medians refer to the eight-category outcome, where lower values indicate more frequent purchasing. BH correction was applied separately to the four binary and four ordinal comparisons. EC = 0/EC = 1 group sizes: BB: 24/140; X: 18/105; Y: 25/156; Z: 35/165. One BB observation had missing EC. Source: own preparation.

**Table 5:** Comparison of unadjusted and adjusted binary logistic regression models.

Specification	Model	Log-Likelihood	Model *df*	AIC	McFadden Pseudo-R^2^	Comparison with Main-Effects Model: Δχ^2^ (*df*), *p*
Unadjusted	Main effects	−444.06	5	900.11	0.026	-
Unadjusted	Main effects + HE × generation	−442.30	8	902.60	0.030	3.51 (3), *p* = 0.319
Unadjusted	Main effects + EC × generation	−437.66	8	893.32	0.040	12.80 (3), *p* = 0.005
Unadjusted	Full model	−435.73	11	895.46	0.045	16.65 (6), *p* = 0.011
Adjusted	Main effects	−438.06	14	906.13	0.040	-
Adjusted	Main effects + HE × generation	−436.38	17	908.76	0.043	3.37 (3), *p* = 0.338
Adjusted	Main effects + EC × generation	−432.00	17	900.00	0.053	12.13 (3), *p* = 0.007
Adjusted	Full model	−430.26	20	902.51	0.057	15.62 (6), *p* = 0.016

Note. Adjusted models additionally control for gender, employment status, household size, and self-assessed financial situation. Comparisons are made against the main-effects model within the same adjustment specification. *n* = 668. Source: own preparation.

**Table 6:** Unadjusted and adjusted generation-specific effects of HE and EC in the binary logistic regression model.

Predictor	Generation	Unadjusted B	Unadjusted SE	Unadjusted OR [95% CI]	*p*	Adjusted B	Adjusted SE	Adjusted OR [95% CI]	*p*
HE	BB	−0.031	0.369	0.97 [0.47, 2.00]	0.933	−0.038	0.373	0.96 [0.46, 2.00]	0.920
HE	X	0.369	0.394	1.45 [0.67, 3.13]	0.349	0.302	0.401	1.35 [0.62, 2.97]	0.452
HE	Y	−0.454	0.313	0.63 [0.34, 1.17]	0.147	−0.498	0.317	0.61 [0.33, 1.13]	0.116
HE	Z	0.269	0.291	1.31 [0.74, 2.32]	0.355	0.204	0.298	1.23 [0.68, 2.20]	0.494
EC	BB	1.275	0.656	3.58 [0.99, 12.94]	0.052	1.272	0.659	3.57 [0.98, 12.99]	0.054
EC	X	1.109	0.671	3.03 [0.81, 11.30]	0.099	1.164	0.676	3.20 [0.85, 12.05]	0.085
EC	Y	0.615	0.457	1.85 [0.76, 4.53]	0.179	0.638	0.463	1.89 [0.76, 4.69]	0.168
EC	Z	−0.827	0.402	0.44 [0.20, 0.96]	0.040	−0.763	0.410	0.47 [0.21, 1.04]	0.063

Note. OR > 1 indicates higher odds of frequent online food purchasing. Adjusted estimates control for gender, employment status, household size, and self-assessed financial situation. Source: own preparation.

**Table 7:** Comparison of unadjusted and adjusted ordinal logistic regression models.

Specification	Model	Log-Likelihood	Model *df*	AIC	Comparison with Main-Effects Model: Δχ^2^ (*df*), *p*
Unadjusted	Main effects	−1273.58	5	2571.16	-
Unadjusted	Main effects + HE × generation	−1272.19	8	2574.38	2.78 (3), *p* = 0.427
Unadjusted	Main effects + EC × generation	−1266.79	8	2563.57	13.58 (3), *p* = 0.004
Unadjusted	Full model	−1265.02	11	2566.05	17.11 (6), *p* = 0.009
Adjusted	Main effects	−1266.35	14	2574.70	-
Adjusted	Main effects + HE × generation	−1264.89	17	2577.79	2.91 (3), *p* = 0.406
Adjusted	Main effects + EC × generation	−1259.97	17	2567.93	12.77 (3), *p* = 0.005
Adjusted	Full model	−1258.20	20	2570.40	16.29 (6), *p* = 0.012

Note. Adjusted models additionally control for gender, employment status, household size, and self-assessed financial situation. The proportional-odds assumption was not fully satisfied; therefore, ordinal results should be treated as sensitivity analyses. Source: own preparation.

**Table 8:** Simple effects of HE and EC in the ordinal logistic regression model.

Predictor	Generation	Unadjusted B	Unadjusted SE	Unadjusted OR [95% CI]	*p*	Adjusted B	Adjusted SE	Adjusted OR [95% CI]	*p*
HE	BB	−0.008	0.285	0.99 [0.57, 1.73]	0.977	0.047	0.287	1.05 [0.60, 1.84]	0.870
HE	X	−0.328	0.319	0.72 [0.39, 1.35]	0.305	−0.299	0.322	0.74 [0.39, 1.39]	0.353
HE	Y	0.295	0.276	1.34 [0.78, 2.31]	0.286	0.340	0.279	1.40 [0.81, 2.43]	0.223
HE	Z	−0.349	0.266	0.71 [0.42, 1.19]	0.189	−0.302	0.270	0.74 [0.44, 1.25]	0.263
EC	BB	−0.773	0.377	0.46 [0.22, 0.97]	0.040	−0.794	0.384	0.45 [0.21, 0.96]	0.039
EC	X	−0.543	0.422	0.58 [0.25, 1.33]	0.198	−0.598	0.424	0.55 [0.24, 1.26]	0.158
EC	Y	−0.399	0.372	0.67 [0.32, 1.39]	0.284	−0.413	0.371	0.66 [0.32, 1.37]	0.266
EC	Z	1.003	0.367	2.73 [1.33, 5.59]	0.006	0.934	0.372	2.54 [1.23, 5.27]	0.012

Note. Because higher outcome values indicate less frequent purchasing, OR > 1 indicates a higher probability of belonging to a less-frequent purchasing category. Adjusted estimates control for gender, employment status, household size, and self-assessed financial situation. The proportional-odds assumption was not fully satisfied; therefore, this model should be treated as a sensitivity analysis. Source: own preparation.

**Table 9:** Summary of direct and moderating effects after multiple-testing correction and multivariable adjustment.

Variable	Separate Direct *p*BH	Separate Interaction *p*BH	Fully Adjusted Direct *p*BH	Fully Adjusted Interaction *p*BH	Final Interpretation
Saving_Orientation	B: 0.385 O: 0.326	B: 0.104 O: 0.068	B: 0.937 O: 0.649	B: 0.253 O: 0.206	Nominal interaction in separate models; not robust after BH correction or full adjustment.
Budget_Control	B: 0.517 O: 0.452	B: 0.406 O: 0.616	B: 0.940 O: 0.904	B: 0.631 O: 0.778	No evidence of a direct or generation-specific association.
Deferred_Purchase	B: 0.596 O: 0.709	B: 0.744 O: 0.781	B: 0.940 O: 0.649	B: 0.691 O: 0.778	No evidence of a direct or generation-specific association.
Expense_Tracking	B: 0.225 O: 0.215	B: 0.175 O: 0.446	B: 0.626 O: 0.494	B: 0.253 O: 0.510	No robust evidence of a direct or generation-specific association.
Price_Comparison	B: 0.114 O: 0.093	B: 0.814 O: 0.810	B: 0.421 O: 0.474	B: 0.709 O: 0.799	Nominal direct association in separate models; not robust after BH correction or full adjustment.
Shopping_Planning	B: 0.385 O: 0.381	B: 0.151 O: 0.256	B: 0.940 O: 0.904	B: 0.253 O: 0.487	Nominal binary interaction only in the separate model; not robust after BH correction or full adjustment.
Careful_Product_Choice	B: 0.114 O: 0.104	B: 0.247 O: 0.551	B: 0.421 O: 0.474	B: 0.312 O: 0.778	Nominal direct association in separate models; not robust after BH correction or full adjustment.

Note. B = binary logistic model; O = ordinal logistic model; *p*BH = Benjamini–Hochberg-adjusted *p* value. Fully adjusted direct-effect models include all seven economic indicators, generation, gender, employment status, household size, and self-assessed financial situation. Fully adjusted interaction tests add the relevant indicator × generation block. *n* = 645. No economic result remained statistically significant after both full adjustment and BH correction. Source: own preparation.

## Data Availability

The original contributions presented in this study are included in the article. Further inquiries can be directed to the corresponding author.

## Appendix A

**Table A1:** Full sociodemographic-adjusted binary logistic regression model.

Predictor	B	SE	OR	95% CI for OR	*p*
Intercept	−2.220	0.704	0.109	[0.03, 0.43]	0.002
Generation X	0.226	0.936	1.254	[0.20, 7.86]	0.809
Generation Y	1.608	0.795	4.992	[1.05, 23.74]	0.043
Generation Z	2.640	0.751	14.019	[3.22, 61.06]	<0.001
HE	−0.038	0.373	0.963	[0.46, 2.00]	0.920
EC	1.272	0.659	3.568	[0.98, 12.99]	0.054
HE × Generation X	0.339	0.546	1.404	[0.48, 4.10]	0.534
HE × Generation Y	−0.460	0.490	0.631	[0.24, 1.65]	0.348
HE × Generation Z	0.242	0.476	1.273	[0.50, 3.23]	0.611
EC × Generation X	−0.108	0.944	0.898	[0.14, 5.71]	0.909
EC × Generation Y	−0.634	0.805	0.531	[0.11, 2.57]	0.431
EC × Generation Z	−2.035	0.774	0.131	[0.03, 0.60]	0.009
Male	0.120	0.165	1.128	[0.82, 1.56]	0.467
Household size: 2 persons	−0.114	0.263	0.892	[0.53, 1.49]	0.663
Household size: 3 persons	0.234	0.265	1.263	[0.75, 2.13]	0.379
Household size: 4 persons	0.212	0.281	1.236	[0.71, 2.14]	0.450
Household size: 5 or more	−0.108	0.380	0.898	[0.43, 1.89]	0.777
Not employed	0.206	0.237	1.229	[0.77, 1.96]	0.385
Financial situation: very good	0.262	0.255	1.300	[0.79, 2.14]	0.304
Financial situation: sufficient	0.221	0.191	1.247	[0.86, 1.81]	0.247
Financial situation: bad/very bad	−0.755	0.434	0.470	[0.20, 1.10]	0.082

Note. Reference categories: Baby Boomers, women, employed respondents, one-person households, and respondents reporting a good financial situation. HE and EC are coded 0/1. *n* = 668. Source: own calculations.

**Table A2:** Full sociodemographic-adjusted ordinal logistic regression model.

Predictor	B	SE	OR	95% CI for OR	*p*
Generation X	−0.169	0.559	0.845	[0.28, 2.53]	0.763
Generation Y	−1.155	0.533	0.315	[0.11, 0.89]	0.030
Generation Z	−2.114	0.513	0.121	[0.04, 0.33]	<0.001
HE	0.047	0.287	1.048	[0.60, 1.84]	0.870
EC	−0.794	0.384	0.452	[0.21, 0.96]	0.039
HE × Generation X	−0.346	0.429	0.707	[0.31, 1.64]	0.420
HE × Generation Y	0.293	0.399	1.340	[0.61, 2.93]	0.464
HE × Generation Z	−0.349	0.392	0.705	[0.33, 1.52]	0.373
EC × Generation X	0.196	0.569	1.217	[0.40, 3.71]	0.731
EC × Generation Y	0.381	0.532	1.464	[0.52, 4.16]	0.474
EC × Generation Z	1.728	0.533	5.628	[1.98, 16.00]	0.001
Male	−0.095	0.141	0.909	[0.69, 1.20]	0.499
Household size: 2 persons	−0.148	0.221	0.863	[0.56, 1.33]	0.505
Household size: 3 persons	−0.445	0.225	0.641	[0.41, 1.00]	0.048
Household size: 4 persons	−0.447	0.245	0.640	[0.40, 1.03]	0.068
Household size: 5 or more	−0.423	0.324	0.655	[0.35, 1.24]	0.192
Not employed	−0.232	0.201	0.793	[0.53, 1.18]	0.250
Financial situation: very good	−0.342	0.220	0.710	[0.46, 1.09]	0.119
Financial situation: sufficient	−0.158	0.163	0.854	[0.62, 1.18]	0.333
Financial situation: bad/very bad	0.480	0.323	1.615	[0.86, 3.05]	0.138

Note. Reference categories: Baby Boomers, women, employed respondents, one-person households, and respondents reporting a good financial situation. HE and EC are coded 0/1. *n* = 668. Threshold parameters are omitted. Higher outcome values indicate less frequent purchasing. The proportional-odds assumption was not fully satisfied; this model should therefore be treated as a sensitivity analysis. Source: own calculations.

**Table A3:** Direct effects of economic decision indicators in separate and fully adjusted models.

Indicator	Outcome	Separate OR [95% CI]	*p*	*p*BH	Fully Adjusted OR [95% CI]	*p*	*p*BH
Saving_Orientation	Binary	1.27 [0.84, 1.93]	0.254	0.385	1.16 [0.73, 1.84]	0.536	0.937
Budget_Control	Binary	0.86 [0.59, 1.26]	0.444	0.517	1.02 [0.67, 1.55]	0.940	0.940
Deferred_Purchase	Binary	1.15 [0.69, 1.89]	0.596	0.596	0.90 [0.52, 1.55]	0.707	0.940
Expense_Tracking	Binary	1.31 [0.95, 1.81]	0.097	0.225	1.22 [0.86, 1.74]	0.268	0.626
Price_Comparison	Binary	1.69 [1.09, 2.61]	0.019	0.114	1.50 [0.94, 2.40]	0.092	0.421
Shopping_Planning	Binary	1.20 [0.87, 1.65]	0.275	0.385	1.01 [0.71, 1.45]	0.936	0.940
Careful_Product_Choice	Binary	1.53 [1.04, 2.25]	0.033	0.114	1.39 [0.92, 2.11]	0.120	0.421
Saving_Orientation	Ordinal	0.79 [0.56, 1.12]	0.187	0.326	0.84 [0.57, 1.24]	0.387	0.649
Budget_Control	Ordinal	1.15 [0.84, 1.58]	0.388	0.452	0.98 [0.69, 1.39]	0.898	0.904
Deferred_Purchase	Ordinal	0.93 [0.62, 1.39]	0.709	0.709	1.18 [0.76, 1.82]	0.464	0.649
Expense_Tracking	Ordinal	0.79 [0.60, 1.04]	0.092	0.215	0.83 [0.61, 1.11]	0.212	0.494
Price_Comparison	Ordinal	0.63 [0.44, 0.91]	0.013	0.093	0.70 [0.48, 1.04]	0.078	0.474
Shopping_Planning	Ordinal	0.86 [0.65, 1.13]	0.272	0.381	0.98 [0.72, 1.33]	0.904	0.904
Careful_Product_Choice	Ordinal	0.70 [0.51, 0.97]	0.030	0.104	0.77 [0.54, 1.09]	0.135	0.474

Note. Separate models include the focal economic indicator and generation. Fully adjusted models include all seven economic indicators, generation, gender, employment status, household size, and self-assessed financial situation. For ordinal models, OR < 1 indicates a higher probability of more frequent purchasing because lower outcome values represent higher purchasing frequency. BH correction was applied separately within each outcome specification. *n* = 645. Source: own calculations.

**Table A4:** Generation-moderation tests for economic decision indicators in separate and fully adjusted models.

Indicator × Generation	Outcome	Separate LR chi2 (*df*)	*p*	*p*BH	Adjusted LR chi2 (*df*)	*p*	*p*BH
Saving_Orientation	Binary	10.48 (3)	0.015	0.104	8.24 (3)	0.041	0.253
Budget_Control	Binary	3.75 (3)	0.290	0.406	2.64 (3)	0.450	0.631
Deferred_Purchase	Binary	1.69 (3)	0.638	0.744	1.91 (3)	0.592	0.691
Expense_Tracking	Binary	6.90 (3)	0.075	0.175	6.06 (3)	0.109	0.253
Price_Comparison	Binary	0.95 (3)	0.814	0.814	1.38 (3)	0.709	0.709
Shopping_Planning	Binary	8.14 (3)	0.043	0.151	6.75 (3)	0.080	0.253
Careful_Product_Choice	Binary	5.46 (3)	0.141	0.247	4.91 (3)	0.179	0.312
Saving_Orientation	Ordinal	11.41 (3)	0.010	0.068	8.99 (3)	0.029	0.206
Budget_Control	Ordinal	2.70 (3)	0.440	0.616	1.72 (3)	0.632	0.778
Deferred_Purchase	Ordinal	1.56 (3)	0.669	0.781	1.57 (3)	0.667	0.778
Expense_Tracking	Ordinal	4.75 (3)	0.191	0.446	4.43 (3)	0.219	0.510
Price_Comparison	Ordinal	0.97 (3)	0.810	0.810	1.01 (3)	0.799	0.799
Shopping_Planning	Ordinal	6.96 (3)	0.073	0.256	5.49 (3)	0.139	0.487
Careful_Product_Choice	Ordinal	3.55 (3)	0.315	0.551	2.60 (3)	0.457	0.778

Note. Each likelihood-ratio test evaluates the three indicator × generation interaction terms jointly. Fully adjusted tests include all seven economic indicators, gender, employment status, household size, and self-assessed financial situation. BH correction was applied separately within each outcome specification. *n* = 645. Source: own calculations.
